# Leading Factors for Weight Gain during COVID-19 Lockdown in a Spanish Population: A Cross-Sectional Study

**DOI:** 10.3390/nu13030894

**Published:** 2021-03-10

**Authors:** Enric Sánchez, Albert Lecube, Diego Bellido, Susana Monereo, María M. Malagón, Francisco J. Tinahones, on behalf of the Spanish Society for the Study of Obesity

**Affiliations:** 1 Endocrinology and Nutrition Department, University Hospital Arnau de Vilanova, Obesity, Diabetes and Metabolism Research Group (ODIM), IRBLleida, University of Lleida, 25198 Lleida, Spain; esanchez@irblleida.cat; 2 Centro de Investigación Biomédica en Red de Diabetes y Enfermedades Metabólicas Asociadas (CIBERDEM), Instituto de Salud Carlos III (ISCIII), 28029 Madrid, Spain; 3 Endocrinology and Nutrition Department, Ferrol University Hospital Complex (CHUF), 15405 A Coruña, Spain; diegobellido@gmail.com; 4 Endocrinology and Nutrition Department, Gregorio Marañón University General Hospital, 28007 Madrid, Spain; smonereoconsulta@gmail.com; 5 Department Cell Biology, Physiology, and Immunology, IMIBIC/University of Cordoba (UCO)/Reina Sofia University Hospital (HURS), 14004 Cordoba, Spain; bc1mapom@uco.es; 6 Centro de Investigación Biomédica en Red de Fisiopatología Obesidad y Nutrición (CIBEROBN), Instituto de Salud Carlos III (ISCIII), 28029 Madrid, Spain; fjtinahones@hotmail.com; 7 Endocrinology and Nutrition Department, Virgen de la Victoria University Hospital, Institute of Biomedical Research of Malaga (IBIMA), University of Malaga, 29010 Málaga, Spain

**Keywords:** COVID-19, lifestyle, lockdown, obesity, Spain

## Abstract

The increase in sedentary behaviors during the COVID-19-induced lockdown may have led to a significant weight gain. To investigate this hypothesis, a representative sample of the Spanish adult population comprising 1000 subjects was enrolled in a cross-sectional study between 26 May and 10 June 2020. Computer-assisted telephone interviews were conducted consisting of 29 questions on the topic of lifestyle habits during the lockdown. The cohort comprised 51.5% women and 51% overweight or obese subjects and had a mean age of 50 ± 18 years. Of the respondents, 44.5% self-reported weight gain during the lockdown; of these, 58.0% were women, 69.9% had previous excess weight, 44.7% lived with a relative who also gained weight, and 73.5 experienced increased appetite. Further, an increased consumption of energy-dense products was found relative to respondents who did not gain weight (*p* ≤ 0.016 for all). Additionally, respondents were unaware that obesity is a poor prognostic factor for COVID-19 infection, lived in smaller flats, and had a lower level of education and lower monthly income. The factors independently associated with weight gain were female gender, previous overweight or obesity, lack of food care, increased appetite, and increased consumption of sugar-sweetened beverages, alcoholic beverages, and snacks (*p* ≤ 0.023 for all). Should another lockdown be mandated, extra caution is warranted to prevent weight gain.

## 1. Introduction

In late 2019, a cluster of patients with pneumonia of unknown etiology was reported in Wuhan, China [1]. At the beginning of 2020, the World Health Organization reported that Chinese authorities had attributed the outbreak to a novel disease (coronavirus disease 2019, or COVID-19) caused by severe acute respiratory syndrome coronavirus 2 (SARS-CoV-2) [2]. COVID-19 has reached global pandemic status, with more than 85 million cases and 1.8 million deaths worldwide in 2020 alone [3]. In Europe, Spain has been one of the most affected countries [4]. 

To date, a substantial corpus of information on COVID-19 has been amassed. The virus has been found to be capable of permeating an entire country within a single month [5]. Various clinical manifestations of COVID-19 have been described, from mild or asymptomatic infection to severe life-threatening disease [6]. Furthermore, the influence of obesity on the severity of COVID-19—which was initially contested—has since been empirically confirmed [7,8,9]. Indeed, a high incidence of obesity has been reported among patients admitted to intensive care for COVID-19 [10,11]. Similarly, obesity has already been established as an independent risk factor in other viral infections such as H1N1 [12]. 

In a study where anthropometric measurements were recorded within participants’ homes in Spain by trained observers, the prevalence of overweight and obesity was found to reach 60.9% [13]. Under Article 116.2 of Spanish Constitution, the Government of Spain declared a state of emergency from March 14 to 21 June 2020 due to the COVID-19 pandemic. This law mandated a lockdown and thereby limited the movement of people. The consequent increase in sedentary behaviors may have repercussions on health [14,15]. Despite the wealth of literature to date on COVID-19, few studies have assessed the short- to medium-term health consequences of COVID-19 lockdown in a Spanish population [16,17,18]. 

Therefore, we aimed to assess self-perceived weight changes during the COVID-19 lockdown in a Spanish sample via computer-assisted telephone interviewing (CATI). We also explored the main sociodemographic characteristics of the participants according to weight gain, as well as the impact of lifestyle modifications during lockdown on weight evolution.

## 2. Materials and Methods

### 2.1. Participants

This cross-sectional observational study enrolled 1000 individuals between 26 May and 10 June 2020, in a representative adult Spanish population sample according to sex, age, and region (excluding Ceuta and Melilla). Depending on the size of the region, population ranged from 1.1% from Navarra to 20.7% from Andalucía. Quota sampling was used, which is a non-probability sampling method. Subjects were classified into one of two groups: the former comprised subjects who self-reported having maintained or lost weight during lockdown, while the latter comprised those who reported weight gain. Furthermore, participants reporting weight gain were classified according to a weight gain ≤ 3 kg or a weight gain of ≥3 kg.

### 2.2. Computer-Assisted Telephone Interviewing (CATI)

The development of CATI was carried out in accordance with the ISO 20252/2012 standard of the Spanish Association for Standardization and Certification for market studies and opinion through the 40 dB company. This law sets a common standard of quality for market investigation globally, which helps agencies to grow and expand. CATI follows the excellence and standards for best practices in survey research [19]. All questions were read over the telephone by the interviewer, and the answers were directly documented in the electronic system. The interviews averaged 8 min (5–16 min) in duration, and no data transformation was made once answers were recorded. A total of 10,924 telephone calls were placed to obtain 1000 complete surveys, which accounts for a relatively poor survey completion rate of 9.2% [20]. 

### 2.3. Outcomes

Our CATI included 29 closed questions about sociodemographic characteristics, weight progression, lifestyle and nutritional habits, and self-weight concern during the COVID-19 lockdown (Table 1). Data on weight and height were obtained through self-report at the time of the interview. From these data, BMI was calculated, and respondents were classified according to the WHO classification [21]. No data about participants’ weight prior the lockdown were obtained. We included a reference group (“no weight gain”) comprising participants who maintained their weight combined with those who lost weight. Lastly, participants were not asked whether they had been infected with COVID-19.

### 2.4. Statistical Analysis

The normal distribution of the variables was evaluated using the Shapiro–Wilk test. Given its normal distribution, quantitative data are expressed as mean ± standard deviation or percentage. Comparisons between the two groups were made using the Student’s *t*-test for quantitative variables and the Pearson’s chi-squared test for categorical variables. A sample size of 1000 subjects reached a statistical power of 95.5% and a 3.1% margin of error for detection of true differences among the variables, with a false discovery rate of 0.001. With 1000 individuals, this study was also able to estimate all coefficients required to develop multivariable logistic prediction models with 21 variables. Therefore, a multivariable logistic regression model for the presence of weight gain was developed while adjusting for the elements that present significant differences between the two groups. The calibration and discrimination of the model was evaluated using the Chi-square goodness of fit test and the area under the receiver operating characteristic (ROC) curve, correspondingly. All statistical tests were two-sided, and statistical significance was set at *p* ≤ 0.05. All statistical analyses were performed using SSPS statistical package (IBM SPSS Statistics for Windows, Version 25.0. Armonk, NY, USA). The reporting abides by the Strengthening the Reporting of Observational studies in Epidemiology (STROBE) STROBE guidelines for cross-sectional studies [22].

### 2.5. Statement of Ethics 

This work was conducted ethically in accordance with the Declaration of Helsinki. This study was also conducted following the ethical standards outlined by The International Chamber of Commerce (ICC)/European Society for Opinion and Marketing Research (ESOMAR) international code on market, opinion, and social research and data analytics. 

## 3. Results

The Spanish sample, 51.5% of whom were female, had a median age and self-reported BMI of 51 ± 18 years and 25.3 ± 3.9 kg/m^2^, respectively (Table 2). Furthermore, 49.8% of respondents were classified as having excess weight (39.9% overweight combined with 9.9% obese). Nevertheless, only 28.8% of respondents reported a self-perception of having excess body weight. 

Additionally, 44.5% of participants reported having gained weight during the lockdown (32.7% increased by <3 kg; 11.8% increased by >3 kg). On the other hand, 49.1% and 6.4% of participants maintained or lost weight during the same period, respectively. Data regarding the main factors related to weight progression during this period are also displayed in Table 2, showing the existence of two well-differentiated populations not only in their baseline anthropometric characteristics but also in their attitudes toward food and physical activity. The participants who gained weight predominantly showed a significantly higher BMI (26.6 ± 3.6 vs. 24.2 ± 3.7, *p* < 0.001) and a higher prevalence of excess weight (69.9% vs. 35.9%, *p* < 0.001). In addition, although they were concerned about their weight, a lower percentage of subjects who gained weight were under medical supervision regarding diet and physical activity (39.1% vs. 51.1%, *p* = 0.016), and a lower percentage of them also considered the lockdown period an opportunity to follow a healthy lifestyle less frequently (30.1% vs. 44.5%, *p* < 0.001). 

When the participants were asked about their knowledge of excess body weight as a risk factor for the prognosis of COVID-19, 48.8% were unaware. However, this proportion increased among those under 25 years of age (67.3%), those who gained more than 3 kg during lockdown (70.7%), and especially in those with a monthly income less than €1000 (80.5%). 

The results also revealed an unequal distribution of unhealthy eating habits during lockdown between groups. Although only 18.0% of respondents reported increased snacking between meals, this rate grew to 30.0% among participants living in houses with an area < 50 m^2^, to 44.4% in those aged 18–24 years and 70.6% in those in the group with no income or <€1000 per month. Furthermore, participants who gained weight reported increased consumption of sugar-sweetened and alcoholic beverages (71.0% vs. 23.1%, *p* < 0.001), bakery products (73.0% vs. 24.7%, *p* < 0.001), ready-to-eat foods (32.2% vs. 10.6%, *p* < 0.001), and red meat (35.3% vs. 19.3%, *p* < 0.001) relative to participants who maintained their weight; inactivity was also significantly higher in the former group (Table 2). Stratified analysis by the amount of weight gain was also performed (Table 3).

The main reason reported by the participants who gained weight (53.9%) during the lockdown was the combination of greater food intake and less physical activity. Furthermore, 73.5% of respondents confessed to having experienced greater and more frequent sensations of hunger than they did before the lockdown. These respondents also had an increased risk ratio of weight gain during the lockdown of 3.8 (95% CI 3.2–4.5), with anxiety as the top factor related with this perception of increased hunger.

**Figure 1:**
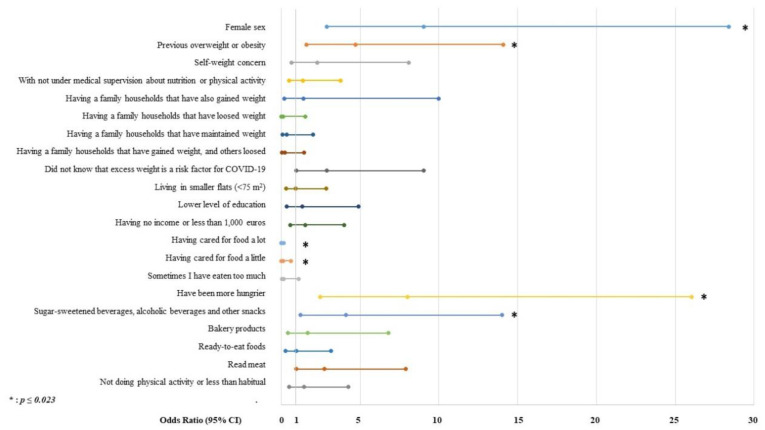
Multivariable logistic regression model for weight gain during the COVID-19 lockdown in Spain.

The multivariate logistic regression model (Figure 1) showed that the independent factors associated with weight gain during the lockdown due to COVID-19 in Spain were female sex (OR 9.04 (95% CI 2.87–28.4), *p* < 0.001), the presence of previous overweight or obesity (4.70 (1.60–14.10), *p* < 0.001), the development of the sensation of being hungrier (8.01 (2.46–26.04), *p* < 0.001), and increased consumption of sugary drinks, alcoholic beverages and snacks (4.11 (1.21–13.99), *p* = 0.023). On the other hand, taking care of food (“I place high importance on the quality of my food” and “I place a little importance on the quality of my food”) appeared to be a protective factor against weight gain during the lockdown (*p* < 0.001). The Chi-squared goodness of fit test for the entire model was 0.184, with an area under the ROC curve of 0.752 (0.719–0.806; *p* < 0.001). Finally, cluster analysis identified three groups of participants who experienced weight gain during the lockdown period. The baseline characteristics of each group are displayed in Table 4.

## 4. Discussion

The results of our study suggest that the lockdown brought about by the COVID-19 pandemic induced moderate weight gain in nearly half of the Spanish population. Most subjects reported a weight gain of 1–3 kg during the 98-day lockdown. A weight increase <3 kg over a brief period can be considered insignificant given that it accounts for less than 5% of the initial weight. However, weight gain can also engender adverse metabolic consequences such as increased blood glucose, dyslipidemia, high blood pressure, and cardiovascular disease, especially among women [23]. Moreover, a mathematical simulation model has predicted an increase in glycated hemoglobin values after 30 and 45 days of COVID-19 lockdown of 2.3% and 3.7%, respectively [24]. Of particular relevance to COVID-19, obesity has markedly deleterious effects on lung function, contributing significantly to the burden of respiratory disease [25].

Similarly, weight gain is often reported during the holiday seasons, corroborating the closely entwined relationship between obesity and lifestyle [26]. Based on the lifestyle variables that we investigated, we identified that the pre-lockdown presence of overweight or obesity, without caring for food, feeling hungrier during the lockdown, and increased consumption of sugar-sweetened drinks, alcoholic beverages, and snacks were independently related to weight gain. However, the most important factor related to weight gain was found to be the female gender. In our study, a higher percentage of women reported weight gain than did men (58.0 vs. 46.3%, *p* < 0.001). In fact, obesity rates in adults have been reported to be as high as 34.9% in the United States, with a higher prevalence found in women [27]. In Spain, 21.6% of adults are obese, a percentage that increases to 32.1% among women aged over 55 years. Furthermore, 60.9% of the Spanish population have been found to be overweight or obese [13]. The rate of overweight and obesity in our study was 49.8%, nearly 10% lower. These data should not be surprising us, since they are similar to differences reported in studies that evaluated discrepancies between self-reported measurements or those reported by trained observers in the participants’ home, and also similar to the data reported in other populations [28,29].

In our study, female sex was the main risk factor associated with weight gain during the COVID-19 lockdown. In addition, women are responsible for more than 75% of the USD $400 billion attributed to obesity in the United States [30]. The increased rate of obesity among women has been partially attributed to their smaller bodies—and thus lower caloric requirements—relative to typical food portions, as well as to weight gain during pregnancy [31]. Reinforcing the role of gender in weight gain, a recent study analyzed data from 50,019 subjects (57.6% female) over 10 years of follow-up in Spain. This study found that young women with a lower BMI were at higher risk of increasing their BMI by more than 2 points during the follow-up period [32]. Therefore, greater priority should be placed on women as a target group for anti-obesity policies.

Although nearly half of the respondents in our sample reported a BMI exceeding 25 kg/m^2^, only 28.8% of participants reported the self-perception of being overweight. This result corroborates those of a recent study promoted by the Spanish Society for the Study of Obesity, which concluded that Spanish culture holds a poor impression of obesity [33]. Differences between objectively recorded and self-reported BMI have been firmly established; thus, self-reported data should be interpreted with caution [34]. Over-reporting height and under-reporting weight and BMI are common in adult subjects [35]. Consequently, the misjudgment of overweight prevalence and weight gain during lockdown may be even greater than our data suggest.

The combination of increased food intake and decreased physical activity was the main explanation cited for weight gain during Spain’s COVID-19 lockdown. With respect to food intake, the Spanish Ministry of Agriculture, Fisheries and Food found that consumption of wine, beer, and other alcoholic beverages, in addition to chocolate, bakery products, snacks, and nuts, increased by more than 50% during the same period [36]. All of these calorie-dense products are likely to have contributed to maintaining and even surpassing pre-COVID-19 energy consumption patterns. 

Our results showed that the consumption of sugar-sweetened beverages, alcoholic beverages, and snacks was strongly associated with weight gain. Given that 1 g of alcohol provides 7.1 kcal (29 kJ) of energy, it is of no surprise that prospective and longitudinal studies show that excessive alcohol consumption is associated with weight gain [37,38,39]. As such, it is necessary to reconsider promoting tax scenarios for the most energy-dense products in order to meaningfully improve industry and consumers behaviors [40,41]. In terms of physical activity, sedentary behavior is like to have decreased the basal metabolic correction factor by 10–50% [42]. Therefore, performing home-based exercises is a behavioral strategy that is strongly encouraged to alter sedentary comportment [43].

Taking care of food was the only protective factor against weight gain in our study. Weight control is known to depend on the ability of individuals to modify their behaviors, which appears to be at odds with our finding of an increased sense of hunger during the lockdown [44]. It has recently been reported that self-quarantine can lead to depression, post-traumatic stress disorder, and lifestyle behaviors that lead to increased rates of obesity [45,46]. Zachary et al. concluded that individuals must alter their coping mechanisms against stress in order to prevent weight gain during self-quarantine [47]. In our study, 73.5% of the participants in the group that gained weight who reported being hungrier during the lockdown increased to 84.7% among those who gained more than 3 kg. Thus, combating this feeling of hunger goes beyond keeping tempting foods at bay and includes understanding how sleep, physical activity, and stress play key roles in the perception of hunger and satiety.

Furthermore, only 51.2% of participants were aware that overweight is a risk factor for a poor prognosis after COVID-19 infection [7,8]. This percentage was even lower among young people and lower social classes, decreasing to 19.5% among those with a monthly income of <€1000. Therefore, better education is warranted about the increased risk of excess body weight in COVID-19 infection in the general population—and especially in the most disadvantaged—as a preventative measure to help to combat weight gain. It has been suggested that among the pathophysiological mechanisms of this relationship in obesity are decreased expiratory reserve volume and functional capacity of the respiratory system. In addition, in subjects with abdominal obesity, lung function has been observed to be even more impaired when in supine position, due to limited diaphragm movement, thereby facilitating respiratory failure [48]. Another proposed pathophysiological mechanism is chronic low-grade inflammation [49]. In this regard, the increase in the concentration of inflammatory cytokines associated with obesity may be exacerbated by COVID-19 [50]. 

Our research has some limitations that should be highlighted, especially regarding methodology. The first limitation is related to the system chosen for data collection; the subjective perception of overweight and obesity is often associated with significant bias [28,29]. For self-reported data in a cross-sectional survey conducted in the community of Vara, Sweden, the sensitivity of obesity was 70% in men and 82% in women [29]. Similarly, the Spanish Society for the Study of Obesity found that only 17.7% of obese people in Spain perceived themselves as obese [27]. However, the prevalence of excess weight in our study shows the expected decrease of 10% compared to population-based studies, supporting the generalizability of our findings. The second limitation is that participants were not asked whether they had been infected by COVID-19, and consequently, data could not be extracted on the possible two-way impact between excess weight and COVID-19 infection. The third limitation is that the survey used was not previously validated and was not administered by a healthcare professional. However, we are pioneering the remote acquisition of data through online channels, such as CATI [27]. Such tools have come to stay, especially in light of the COVID-19 pandemic. The fourth limitation is that no pre-lockdown weight data were available, and therefore, a subtraction analysis could not be performed. Finally, the survey completion rate was low due to the 10,924 calls required to obtain the statistically robust 1000 complete survey. However, although a low response rate can introduce significant bias when interpreting results, many estimates based on telephone surveys with fairly low response rates coincide with estimates from surveys with higher response rates [20].

In conclusion, we evaluated the leading factors related to weight gain during COVID-19 lockdown in Spain. Should another lockdown be required in the future, overweight or obese women are encouraged to focus their efforts to avoid weight gain. In addition, society at large is encouraged to implement measures to reduce the increased feelings of hunger during lockdown periods and to reduce the consumption of sweetened and alcoholic beverages and snacks. 

## Figures and Tables

**Table 1:** The full computer-assisted telephone interviewing (CATI).

Sex, Age, Region, Weight and Height
1.-Are you worried about your weight? Yes/No
2.-Thinking about before you entered lockdown, would you say that you were overweight? Yes/No
3.-Were you under medical supervision regarding diet or a physical activity regime before lockdown? Yes/No
4.-Have you followed the medical advice provided regarding diet and physical activity? Yes/No
5.-If “no”: Why have you not followed it? It was not possible to follow up with my dietitian, primary care physician, or endocrinologist, etc./It was difficult to follow due to anxiety, stress, etc./It was difficult to purchase healthy products/It was difficult to go to the gym/Other reason
6.-The professional consulted for weight loss: Pharmacist/Primary care physician/Endocrinologist/Dietitian/Friend/Internet
7.-Do you think that lockdown has been an opportunity to follow a healthy lifestyle? Yes/No
8.-How did your weight change during lockdown? I lost weight/I maintained my normal weight/I gained weight (<3 kg)/I gained weight (≥3 kg)
9.-If you gained weight, what was the reason? Greater food intake/Less physical activity/Both factors equally
10.-What happened with the rest of the household’s members? They gained weight/They lost weight/They maintained normal weight/Different weight progressions/I live alone
11.-How many people do you live with? Alone/1/2/3/>3
12.-Did you know that excess weight is a risk factor for COVID-19? Yes/No
13.-How many square meters does your house have? < 50 m^2^/50–75 m^2^/75–150 m^2^/> 150 m^2^
14.-What educational level have you achieved? Primary studies or lower/Secondary studies/University or higher
15.-What is your monthly income? None or <€1000/€1000–2000/€2000–3000/€3000–4000/>€4000
16.-Which of the following statements is most accurate? I place high importance on the quality of of my food/I place a little importance on the quality of my food/Sometimes I have eating so much/I do not care about the quality of my food
17.-Did you have been hungrier? Yes/No
18.-Did you increase consumption of sugar-sweetened beverages, alcoholic beverages, or other snacks? Yes/No
19.-Did you increase consumption of bakery products? Yes/No
20.-Did you increase consumption of ready-to-eat- foods? Yes/No
21.-Did you increase consumption of red meat? Yes/No
22.-Did you increase consumption of white meat? Yes/No
23.-Did you increase consumption of fish? Yes/No
24.-What was the reason? I had anxiety/I was bored/I was near the fridge/All of the above
25.-How many times have you eaten on average per day? 2/3/> 3/I have been eating continuously
26.-Did you regularly practice physical activity? Yes/no
27.-If you practiced physical activity, would you say that…? I practiced less than habitual/Equal/More than habitual
28.-How many hours did you spend sitting on an average day, including when completing work or schoolwork? 1–2 h/3–4 h/5–7 h/More than 7 h
29.-How many hours did you spend sitting and watching screens on an average day? <1/1–3 h/3–4 h/5–7 h/>7 h

**Table 2:** Interview results regarding the main attitudes held the lockdown period according to weight progression.

	All Population (*n* = 1000)	No Weight Gain (*n* = 555)	Weight Gain (*n* = 445)	*p*
Female, *n* (%)	515 (51.5)	257 (46.3)	258 (58.0)	<0.001
Age (year)	51 ± 18	51.2 ± 18.2	50.9 ± 16.8	0.733
Body mass index, (kg/m^2^)	25.3 ± 3.9	24.2 ± 3.7	26.6 ± 3.6	<0.001
Overweight or obesity, *n* (%)	498 (49.8)	199 (35.9)	311 (69.9)	<0.001
Worried about their weight, *n* (%)	523 (52.3)	182 (32.8)	341 (76.6)	<0.001
Self-perception of be overweight, *n* (%)	288 (28.8)	87 (15.7)	201 (45.2)	<0.001
Under medical supervision for diet or physical activity, *n* (%)	458 (45.8)	284 (51.1)	174 (39.1)	0.016
Not following medical advice, *n* (%)	60 (44.9)	10 (20.8)	50 (62.5)	<0.001
The lockdown is an opportunity to follow a healthier lifestyle, *n* (%)	381 (38.1)	247 (44.5)	134 (30.1)	<0.001
Having family members who also gained weight, *n* (%)	261 (26.1)	62 (11.2)	199 (44.7)	<0.001
Did not know that excess weight is a risk factor for COVID-19, *n* (%)	488 (48.8)	226 (40.7)	262 (58.9)	<0.001
Living in housing of <50 m^2^, *n* (%)	335 (33.5)	178 (32.1)	157 (35.3)	<0.001
Primary studies or less? *n* (%)	263 (26.3)	138 (24.8)	125 (28.1)	0.002
No income or < €1.000, *n* (%)	333 (33.3)	155 (27.9)	178 (40.0)	<0.001
Without worrying about food, *n* (%)	147 (14.7)	11 (2.0)	136 (30.6)	<0.001
Have been hungrier than before the lockdown, *n* (%)	421 (42.1)	94 (16.9)	327 (73.5)	<0.011
Increased consumption of sugar-sweetened and alcoholic beverages, and other snacks, *n* (%)	444 (44.4)	128 (23.1)	316 (71.0)	<0.001
Increased consumption of bakery products, *n* (%)	462 (46.2)	137 (24.7)	325 (73.0)	<0.001
Increased consumption of ready-to-eat foods, *n* (%)	220 (22.0)	59 (10.6)	161 (32.2)	<0.001
Increased consumption of red meat, *n* (%)	264 (26.4)	107 (19.3)	157 (35.3)	<0.001
Performed intermittent or irregular physical activity, *n* (%)	321 (56.1)	173 (45.9)	148 (75.9)	<0.001
Spent >5 h/day sitting watching screens, *n* (%)	540 (54.0)	231 (41.6)	309 (69.4)	<0.001
Eating continuously, *n* (%)	179 (17.9)	19 (3.4)	160 (36.2)	<0.001

**Table 3:** Significant differences in respondents who self-reported weight gain according to the amount of weight gained.

	Weight Gain < 3 kg (*n* = 327)	Weight Gain ≥ 3 kg (*n* = 118)	*p*
Body mass index, (kg/m^2^)	26.2 ± 3.7	27.8 ± 3.2	<0.001
Overweight or obesity, *n* (%)	212 (64.8)	99 (83.9)	<0.001
Worried about their weight, *n* (%)	236 (72.2)	109 (89.0)	<0.001
Self-perception of be overweight, *n* (%)	130 (39.8)	71 (60.2)	<0.001
Under medical supervision for diet or physical activity, *n* (%)	59 (49.4)	21 (29.6)	0.029
Not following medical advice, *n* (%)	33 (55.9)	17 (81.0)	0.042
The lockdown is an opportunity to follow a healthier lifestyle, *n* (%)	208 (63.6)	103 (87.3)	<0.001
Did not know that excess weight is a risk factor for COVID-19, *n* (%)	179 (54.7)	83 (70.3)	<0.001
Without worrying about food, *n* (%)	75 (22.9)	61 (51.7)	<0.001
Have been hungrier than before the lockdown, *n* (%)	227 (69.4)	100 (84.7)	<0.001
Increased consumption of sugar-sweetened and alcoholic beverages, and other snacks, *n* (%)	211 (64.5)	105 (89.0)	<0.001
Increased consumption of bakery products, *n* (%)	223 (68.2)	102 (86.4)	<0.001
Increased consumption of ready-to-eat foods, *n* (%)	103 (31.5)	58 (49.2)	<0.001
Spent >5 h/day sitting watching screens, *n* (%)	28 (63.6)	101 (85.6)	<0.001
Eating continuously, *n* (%)	97 (29.7)	64 (54.2)	<0.001

**Table 4:** Cluster analysis to identify participants at risk for weight gain during lockdown.

	Group 2 (*n* = 177)	Group 3 (*n* = 134)	Group 1 (*n* = 134)	*p*
Female, *n* (%)	177 (100)	81 (60.4)	0 (0)	<0.001
Age (year)	54.1 ± 16.6	45.6 ± 17.1	52.1 ± 15.5	<0.001
Body mass index (kg/m^2^)	28.1 ± 2.9	22.7 ± 1.4	28.6 ± 3.0	<0.001
Overweight or obesity, *n* (%)	177 (100)	0 (0)	134 (100)	<0.001

## Data Availability

The data that support the findings of this study are available from the corresponding author (Lecube A) and the Spanish Society for the Study of Obesity upon reasonable request.
